# Changes in the DNA methylation pattern of the host male gametophyte of viroid-infected cucumber plants

**DOI:** 10.1093/jxb/erw353

**Published:** 2016-10-03

**Authors:** Mayte Castellano, German Martinez, Maria Carmen Marques, Jordi Moreno-Romero, Claudia Köhler, Vicente Pallas, Gustavo Gomez

**Affiliations:** ^1^Instituto de Biología Molecular y Celular de Plantas (IBMCP), Consejo Superior de Investigaciones Científicas (CSIC)-Universidad Politecnica de Valencia (UPV), CPI, Edificio 8 E, Av. de los Naranjos s/n, 46022 Valencia, Spain; ^2^Department of Plant Biology, Swedish University of Agricultural Sciences and Linnean Center for Plant Biology, SE-750 07 Uppsala, Sweden

**Keywords:** Cucumber, epigenetic inheritance, hop stunt viroid, viroid–plant interactions, viroid-induced pathogenesis, viroids and DNA methylation.

## Abstract

Eukaryotic organisms exposed to adverse conditions are required to show a certain degree of transcriptional plasticity in order to cope successfully with stress. Epigenetic regulation of the genome is a key regulatory mechanism allowing dynamic changes of the transcriptional status of the plant in response to stress. The *Hop stunt viroid* (HSVd) induces the demethylation of ribosomal RNA (rRNA) in cucumber (*Cucumis sativus*) leaves, leading to increasing transcription rates of rRNA. In addition to the clear alterations observed in vegetative tissues, HSVd infection is also associated with drastic changes in gametophyte development. To examine the basis of viroid-induced alterations in reproductive tissues, we analysed the cellular and molecular consequences of HSVd infection in the male gametophyte of cucumber plants. Our results indicate that in the pollen grain, accumulation of HSVd RNA induces a decondensation of the generative nucleus that correlates with a dynamic demethylation of repetitive regions in the cucumber genome that include rRNA genes and transposable elements (TEs). We therefore propose that HSVd infection impairs the epigenetic control of rRNA genes and TEs in gametic cells of cucumber, a phenomenon thus far unknown to occur in this reproductive tissue as a consequence of pathogen infection.

## Highlight

The *Hop stunt viroid* (HSVd) is associated with changes in the DNA methylation pattern of cucumber pollen that affect rRNA genes and transposable elements, thus opening the door to the possibility that infection could result in epigenetic effects being passed to the next generation.

## Introduction

The maintenance of genome stability is a constant requirement in living organisms. At the same time, however, organisms must ensure that a certain level of genome plasticity is available in order to allow for genome rearrangements and mutations that might introduce beneficial traits to cope with stresses. In the case of plants, their sessile nature means that they are constantly exposed to different biotic and abiotic stresses that very often affect genome stability in both somatic and meiotic cells (Boyko and Kovalchuk, 2011; Zhu *et al.*, 2016). Viroids are pathogenic long non-coding RNAs (lncRNAs), able to infect and systemically invade herbaceous and ligneous plants (Flores *et al.*, 2005; Ding, 2009; Gomez and Pallas, 2013). Constrained by their small (250–400 nts) and non-protein-coding genome, viroids have evolved into versatile nucleic acids that subvert the plant-cell machinery at diverse functional levels in order to guarantee that their life cycle can be completed within the infected host (Ding, 2009). Although some pathogen–host interactions occur without visible plant alterations (latent diseases), viroid infection is frequently associated with phenotypic changes that we recognize as symptoms.

Because these pathogenic RNAs lack protein-coding activity, it was initially assumed that viroid-induced symptoms resulted from a direct interaction between specific structural elements of the viroid RNA genome and certain host factors (proteins or nucleic acids) (Ding, 2009; Navarro *et al.*, 2012). However, in recent years an increasing amount of experimental data has provided evidence for the existence of other potential pathogenic mechanisms, for example the close interplay between viroid-induced pathogenesis and RNA silencing. The initially proposed idea that certain viroid-derived small RNAs (vd-sRNAs) can down-regulate *in trans* host mRNAs to promote expression of symptoms (Papaefthimiou *et al.*, 2001; Wang *et al.*, 2004; Gomez *et al.*, 2009) was experimentally validated for members of both *Pospiviroidae* (Gomez *et al.*, 2008; Eamens *et al.*, 2014; Adkar-Purushothama *et al.*, 2015) and *Avsunviroidae* (Navarro *et al.*, 2012) families. The observation that the *Cucumber mosaic virus* Y-satellite RNA uses a similar mechanism to alter host-gene expression (Shimura *et al.*, 2011; Smith *et al.*, 2011) suggests that this pathogenesis strategy is not exclusive for viroids. At a different functional level, it is also recognized that in addition to viroids, viruses, bacteria, nematodes, and aphids can alter the miRNA (Ruiz-Ferrer and Voinnet, 2009; Garcia and Pallas, 2015) or sRNA-metabolism (Cao *et al.*, 2014) of their host plants.

Recent studies have produced evidence of global changes of the epigenetic regulation of the host-genome upon viroid infection. An examination of the interaction of *Hop stunt viroid* (HSVd) with two different hosts (*Cucumis sativus*, cucumber, and *Nicotiana benthamiana*) showed that viroid-accumulating plants exhibit an increased rRNA transcription rate. This altered transcription was associated with reduced cytosine methylation of rDNA promoter regions, revealing that some (normally silenced) rRNA genes are transcriptionally reactivated during HSVd infection (Martinez *et al.*, 2014; Castellano *et al.*, 2015). However, induction of changes in the host epigenome is not exclusive for viroid infection. Indeed, dynamic changes in host-DNA methylation patterns occur during antibacterial or antiviral defence in rice (Sha *et al.*, 2005), tobacco (Boyko *et al.*, 2007), and Arabidopsis (Dowen *et al.*, 2012; Yu *et al.*, 2013). Furthermore, overexpression of the replication-associated protein (Rep) of a geminivirus has been shown to induce hypomethylation of host DNA in *N. benthamiana* plants (Rodriguez-Negrete *et al.*, 2013). Taken together, these observations support the notion that host-DNA demethylation may be part of a common induced immune response in plants (Alvarez *et al.*, 2010; Zhu *et al.*, 2016).

In viroid–cucumber interactions DNA demethylation has been connected with HSVd recruiting and functionally subverting the host HISTONE DEACETYLASE 6 (HDA6) (Castellano *et al.*, 2016). In Arabidopsis HDA6 confers an epigenetic memory of the silent state (Blevins *et al.*, 2014) and is furthermore involved in the maintenance and *de novo* CG and CHG (where H is A, T or C) methylation of transposable elements (TEs), rRNA genes, and transgenes via its interaction with DNA METHYLTRANSFERASE 1 (MET 1) and the RNA-directed DNA methylation (RdDM) pathway (Aufsatz *et al.*, 2002; Probst *et al.*, 2004; Earley *et al.*, 2010; Liu *et al.*, 2012; Hristova *et al.*, 2015). Viroids are pathogenic, long non-coding RNAs (lncRNAs) that subvert endogenous lncRNA-directed regulatory routes to complete their life cycle in the infected cell (Gomez and Pallas, 2013). Remarkably, endogenous lncRNAs are able to function as epigenetic modulators by binding to chromatin-modifying proteins and recruiting their catalytic activity to specific sites in the genome, thereby modulating chromatin states and impacting on gene expression (Mercer and Mattick, 2013).

HSVd is a polyphagous pathogenic lncRNA that is able to infect a wide range of hosts (including cucumber, grapevine, citrus, plum, and peach) and causes diverse symptoms (Pallas *et al.*, 2003; Sano, 2003). In cucumber, as well as causing plant stunting, HSVd infection induces severe alterations in reproductive organs that are frequently associated with reduced fertility (Singh *et al.*, 2003; Martinez *et al.*, 2008). Although HSVd is poorly transmitted through seeds and pollen, seed transmission may play a role for its survival in certain hosts such as grapevine (Wah and Symons, 1999). The molecular mechanisms underlying the structural and functional alterations in host reproductive organs associated with viroid infection are currently unknown.

Having established that HSVd alters DNA methylation in vegetative cells, in this study we addressed the question as to whether the epigenetic changes induced by viroid infection are also present in the male gametophyte. Pollen grains are known to transmit other members of the *Pospiviroidae* family (Singh, 1970; Kryczyński *et al.*, 1988; Barba *et al.*, 2007; Card *et al.*, 2007). Our results reveal that both HSVd mature forms and vd-sRNAs can be recovered from pollen grains of infected cucumber plants. Moreover, viroid accumulation is associated with increased pollen germination levels and heterochromatin decondensation in the generative nucleus. Analysis of DNA methylation in rDNA and TE repeats reveal a significant reduction in the symmetric cytosine methylation context, which is associated with a transcriptional increase of their RNAs. In summary, our results show that previously observed epigenetic changes in vegetative tissues are maintained in male gametes and are thus passed on to the next generation

## Material and methods

### Plant material

Six cucumber (*Cucumis sativus* cv Marketer) plants were inoculated with *Agrobacterium tumefaciens* strain C58C1 transformed with a binary pMOG800 vector carrying a head-to-tail infectious dimeric HSVd cDNA (Y09352) (Gomez and Pallas, 2006), as previously described (Gomez *et al.*, 2008). Three cucumber plants infiltrated with *A. tumefaciens* strain C58C1 transformed with a binary pMOG800 empty vector were used as a mock-inoculated control. Plants were maintained in growth chambers at 30 °C for 16h with fluorescent light and at 25 °C for 8h in darkness until flowering. Viroid systemic infection in HSVd-inoculated plants was confirmed by dot-blot hybridization (see Supplementary Fig. S1 at *JXB* online). To collect pollen grains, a paintbrush was used to gently brush pollen from the anthers into an Eppendorf tube. This procedure was repeated for approximately 750 and 650 mature flowers recovered from HSVd-infected and control plants respectively, between 80 and 110 d post-infiltration.

### RNA isolation

As described previously (Aparicio *et al.*, 1999), 20mg of pollen grains were suspended in 1.5ml of phosphate saline-Tween polyvinylpyrrolidone buffer (pH 7.4), vigorously shaken for 1min, and centrifuged at 3000rpm (1000 *g*) for 5min. This procedure was repeated three times, followed by an additional washing with 1.5ml of 1% sodium dodecyl sulphate (SDS) to remove particles firmly bound to the pollen grains. Aliquots from the four supernatants were phenol extracted and the aqueous phase was ethanol precipitated and resuspended in sterile water to check for surface contamination of the viroid. Washed pollen was homogenized for total RNA extraction. Total RNA was extracted from pooled pollen grains (~0.1g) recovered from infected and control cucumber plants using the TRI reagent (SIGMA, St. Louis, MO, USA) according to the manufacturer’s instructions. The low-molecular weight RNA (<200 nt) fraction was enriched from total RNA using MIRACLE (miRNA isolation Kit, STRATAGENE) according to the manufacturer’s instructions. Supernatants and washed pollen were analysed for the presence of HSVd by RT-PCR as described by Martinez *et al.* (2010).

### Small RNA library information

The sRNA sequences used in this work were obtained from an sRNAs population recovered from the pollen of mock-inoculated and *Hop stunt viroid*-infected cucumber plants. The libraries were sequenced using a HIseq 2500 system (Illumina Technology).

### Bisulfite conversion and sequencing

Total genomic DNA was extracted from pollen grains (~0.1g) recovered from different infected and healthy cucumber plants (Dellaporta *et al.*, 1983). Bisulfite treatment was performed using the EpiTec Bisulfite kit (Qiagen). The DNA regions to be analysed and their corresponding oligos were determined using the MethPrimer software (http://www.urogene.org/methprimer/) (Li and Dahiya, 2002). Modified DNA was amplified by PCR using Taq DNA polymerase (Promega). The following primers were used to amplify by PCR specific regions of rDNA and TE, respectively: 45s-Fw ATCATAGATTTTTYGAGGGT (position –80 to –61), 45s-Rv ATGACGACRTAAACATCCCAA (position +101 to +121) (according sequence X51542.1); and TE-Fw TAGTTTTTTGAYAGGGGAAATA (position 545 to 566), TE-Rv CATTCATAAACTTRCTTTCTCA (position 760 to 781) (according TE cucumber predicted sequence: cuc_reannotTE.Scaffold000159.7) (Li *et al.*, 2011). The amplicons obtained were cloned using the InsTAclone PCR cloning Kit (Thermo Scientific). We selected for sequencing sixteen to fifty clones (obtained from two independent replicates) from rDNA and TE, respectively, for each analysis in both the HSVd-infected and control pollen.

### RT-PCR analysis

Total RNA, extracted from pollen grains collected from HSVd-infected and control plants, was treated with DNase in order to avoid DNA contamination. Reverse transcription (RT) PCR analysis of serial dilutions (500, 100, and 20ng) of total RNAs obtained from HSVd-infected and control pollen was performed using the SuperScript® III One-Step RT-PCR System with Platinum® Taq DNA Polymerase (Invitrogen) according to the manufacturer’s instructions. The sequence and relative position of the specific primers used to amplify a region ( ~160 nt) of rRNA precursor by RT-PCR are detailed in Supplementary Table 1. RT-PCR conditions were 45 °C/30min, 95 °C/15s, 51 °C/30s, 72 °C/20s (30 cycles).

To amplify the TE transcripts we used the oligos (TE-Dir TAGCTTTCTGACAGGGGAAATACC, and TE-Rv GCATTCATGAACTTGCTTTCTCAGC) flanking a region (~240bp) of an annotated TE cucumber sequence (cuc_reannotTE.Scaffold000159.7) (Li *et al.*, 2011). RT-PCR conditions were 45 °C/30min, 95 °C/15s, 62 °C/30s, 72 °C/15s (30 cycles). The primers Ub-Dir (5′ CACCAAGCCCAAGAAGATC) and Ub-Rev (5′ TAAACCTAATCACCACCAGC) flanking a region (~220 nt) of ubiquitin mRNA (AN: NM-001282241.1) were used to amplify this mRNA as a load control. RT-PCR conditions were 45 °C/30min, 95 °C/15s, 57 °C/20s, 72 °C/20s (27 cycles). Three repetitions of this analysis were performed. To discard the possible amplification of residual genomic rDNA, 100ng of total RNAs were analysed by PCR using the primer pairs (Fw-25s CACCAATAGGGAACGTGAGCTG, Rv-25s GCGCAATGACCAATTGTGCG) flanking a region (~130bp) of 25s-rRNA and TE-Dir–TE-Rv, detailed above (see Supplementary Fig. S2).

### Real-time quantitative PCR assays

Total RNAs were extracted from pollen grains as described above. First-strand cDNA was synthetized by pulsed reverse transcription (Varkonyi-Gasic *et al.*, 2007) using a RevertAid cDNA Synthesis Kit (Thermo Scientific^TM^) as follows. An initial step at 16 °C for 10min was followed by 45 cycles of 16 °C for 2min, 42 °C for 1min and 50 °C for 1s, including a final denaturing step at 85 °C for 5min. qRT-PCR assays were performed using PyroTaq EvaGreen mix Plus (ROX) (CulteK Molecular Bioline) according to the manufacturer’s instructions. Ubiquitin mRNA (AN: NM-001282241.1) was used to normalize samples.

Detection of small RNAs was performed starting from low molecular weight RNA (<200 nt) fractions obtained as described above. Stem-loop-specific reverse transcription for sRNAs detection was performed as previously described by Czimmerer *et al.* (2013) using a RevertAid cDNA Synthesis Kit (Thermo Scientific). Cucumber miR159 with stable expression in both analysed samples according to the sequencing data was used for normalization. All analyses were performed in triplicates on an ABI 7500Fast-Real Time qPCR instrument (Applied Biosystems) using a standard protocol. The efficiency of PCR amplification was derived from a standard curve generated by four five-fold serial dilution points of cDNA mixed from the two samples. Gene expression was quantified by the comparative ΔCt method. The primers used for cDNA synthesis and qRT-PCR are described above or listed in Supplementary Tables S1 and S2.

## Results

### Cucumber reproductive tissues are affected as a consequence of HSVd-infection

**Fig. 1.**
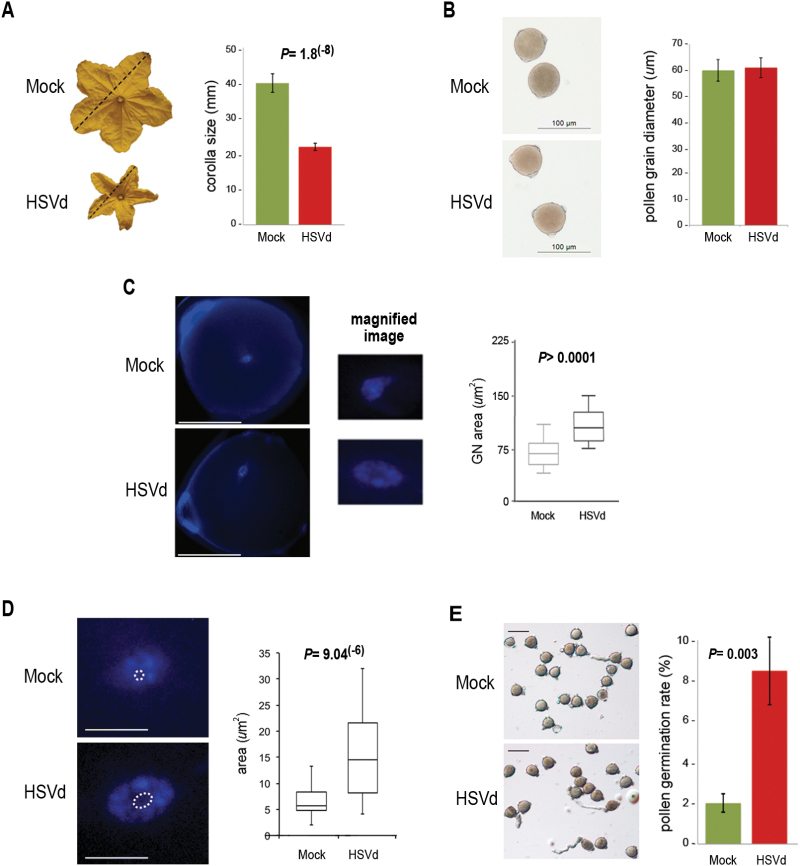
Effects of HSVd infection on cucumber reproductive tissues. (A) Male and female flowers obtained from HSVd-infected and mock-inoculated cucumber plants were measured as indicated by the dashed lines shown on the representative images of male flowers. The graph shows mean values of 750 (HSVd-infected) and 650 (control) flowers. Statistical significance was tested using a paired *t*-test. (B) Micrographs of representative pollen grains recovered from HSVd-infected and mock-inoculated cucumber plants. The graph shows the mean diameter of 200 pollen grains recovered from infected and control plants. No significant differences were observed (paired *t*-test). (C) Representative images of DAPI-stained pollen grains from non-infected and HSVd-infected plants and magnifications of the respective generative nuclei (GN) of the cells. Scale bars = 30 µm. The box-plots show the distribution of the measured areas of the GN of more than 100 pollen grains. Statistical significance was tested using a paired *t*-test with Welch’s correction. (D) Representative images of DAPI-stained generative nuclei of cells. The area corresponding to the nucleolus is highlighted. Scale bars = 150 µm. The box-plots show the distribution of the measured areas of the nucleolus in 32 pollen grains. Statistical significance was tested using a paired *t*-test. (E) Representative images of germinated pollen grains recovered from mock- and HSVd-infected plants. The graph shows the germination rate of infected pollen grains in comparison with control samples. A total of 1200 pollen grains were analysed for each sample in six independent replicates. Statistical significance was tested using a paired *t*-test. In all the figure parts the error bars represent the standard error. Only significant *P* values are shown in the figure. (This figure is available in colour at *JXB* online.)

A characteristic symptom related to HSVd infection is an alteration of fertility that triggers deficiencies in flower size (Sano, 2003; Martinez *et al.*, 2008), fruit quality, and seed viability (Singh *et al.*, 2003; Martinez *et al.*, 2008). In order to determine precisely the level of phenotypic effects induced by HSVd-infection in reproductive organs, we analysed diverse morphological aspects of cucumber flowers. As shown in the Fig. 1A flowers (male and female) obtained from HSVd-infected plants exhibited a significant reduction (close to 50%) of the corolla-size, as previously observed in the HSVd–*N. benthamiana* interaction (Martinez *et al.*, 2008). Morphological studies showed that HSVd did not induce alterations in pollen grain size (Fig. 1B). Study of pollen grains by DAPI staining showed a significant size increase of the generative cell nucleus in infected cells (Fig. 1C). A more detailed analysis revealed that a comparable size increase was also observed in the nucleolus of infected pollen grains (Fig. 1D), suggesting a global alteration of chromatin structure in pollen grains under viroid infection. To determine whether this alteration is associated with physiological changes, we performed germination assays. As shown in Fig. 1E, pollen grains derived from HSVd-infected plants had a higher germination rate than pollen collected from control plants, suggesting that regulatory processes associated with pollen germination are affected during HSVd-infection.

### HSVd accumulates in pollen grains of infected plants

**Fig. 2.**
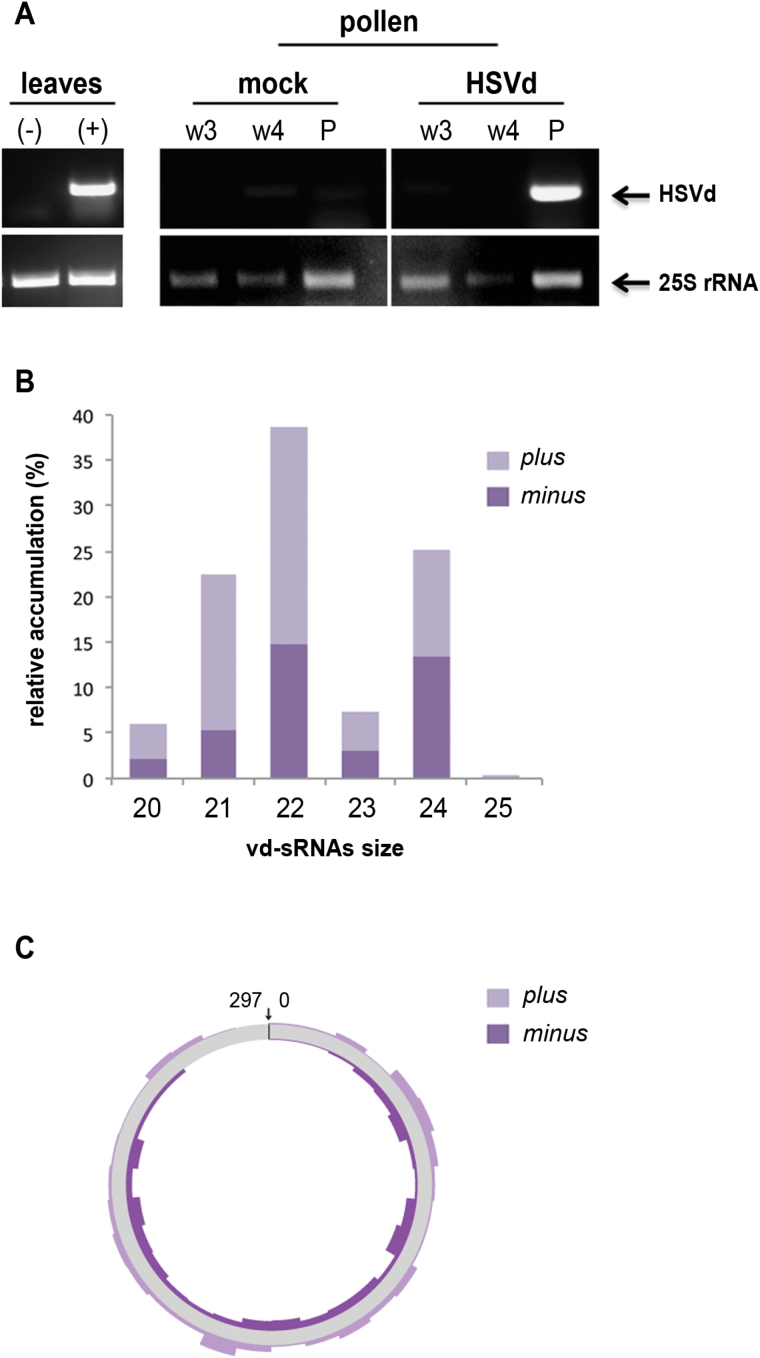
HSVd accumulates in pollen grains of infected cucumber plants. (A) Detection of HSVd RNA by RT-PCR in pollen grains (P) recovered from infected plants (right). Total RNA extracted from successive washing of pollen grains (w3 and w4) was analysed by RT-PCR in order to discard contamination with HSVd RNA of non-pollen-specific plant tissue. Total RNAs extracted from infected (+) and mock-inoculated (-) cucumber leaves were used as RT-PCR controls (left). (B) Size distribution and polarity of canonical (20 to 25 nts) fully homologous total viroid-derived sRNAs (vd-sRNAs) recovered from the infected pollen library. The values on the *y*-axis represent the abundance of vd-sRNAs in the library. (C) The vd-sRNAs were plotted onto a circular sequence of the HSVd RNA, in either sense (plus) or antisense (minus) configuration. The arrow indicates the position of nucleotides 1 and 297 in the circular HSVd RNA. (This figure is available in colour at *JXB* online.)

Having established that HSVd induces structural and functional alterations in the pollen of infected plants, we attempted to determine if viroid molecules could be detected in mature dehiscent pollen grains. RT-PCR assays demonstrated that genomic HSVd RNA accumulated in the pollen grains obtained from infected plants (Fig. 2A), indicating that HSVd is able to invade pollen of infected cucumber plants as has been shown for *Potato spindle tuber viroid* (PSTVd) in potato (Singh *et al.*, 1993) and petunia plants (Matsushita and Tsuda, 2014). We did not detect HSVd on the surface of the pollen grain (Fig. 2A), providing robustness to this affirmation.

Given that in cucumber vegetative tissues genomic HSVd RNA accumulation is associated with the presence of vd-sRNAs (Martinez *et al.*, 2010), we prepared sRNA libraries from pollen grains derived from mock-inoculated and HSVd-infected plants. A total of 4 918 251 and 4 566 866 raw sequences (ranging from 18 to 36 nt) were obtained from HSVd-infected and control pollen grains sRNA libraries, respectively. Sequences ranging from 20 to 25 nts (2 888 088 for infected pollen and 2 523 747 for the control data set) were used for further analysis. When sRNAs recovered from infected and non-infected pollen-grains were analysed by pairwise alignment against the HSVd genome, we observed that a total of 18 900 sequences (0.68%; ranging 20 to 25 nts in length) recovered from the infected pollen were perfectly complementary to HSVd and considered as vd-sRNAs. Importantly, no sequences perfectly matching with HSVd were recovered from the control cucumber pollen, confirming the integrity of the RNA samples. Analysis of polarity distribution indicated that sRNAs derived from the sense strand were slightly biased (60%) in comparison to sRNAs derived from the antisense strand (40%) (Fig. 2B). This vd-sRNA landscape is different to that previously described in cucumber vegetative tissues where HSVd-derived sRNAs of both polarities were recovered at comparable levels (Martinez *et al.*, 2010). Categorized by size, vd-sRNAs were mainly of 22 nt (34.1%), 24 nt (22.1%), and 21 nt (19.9%), whereas vd-sRNAs of 20, 23 and 25 nt amounted to under 6% of the total vd-sRNAs (Fig. 2B). As previously observed in infected leaves, sense and antisense vd-sRNAs spreading along the entire HSVd genome showed a heterogeneous distribution pattern (Fig. 2C). In summary, pollen grains of HSV-infected plants accumulate HSVd RNA and vd-sRNAs.

### The endogenous sRNA profile is altered in HSVd-infected pollen

**Fig. 3.**
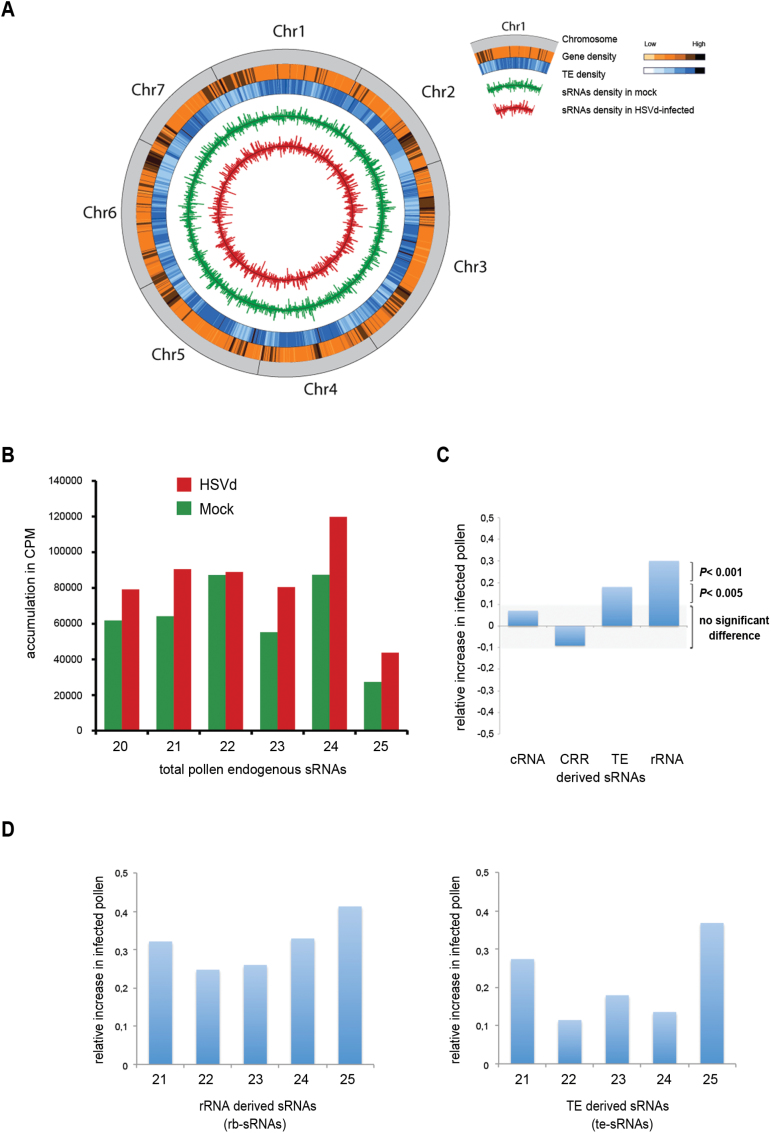
Characterization of the small RNAs recovered from cucumber pollen grains by deep sequencing. (A) Whole-genome distribution of sRNA density in control and HSVd-infected pollen grains. The outermost to innermost tracks depicti: *C. sativus* chromosomes (Chr 1 to 7); heat map of gene density (light = low density, dark = high density); heat map of transposable element density (light = low density, dark = high density); sRNA density in mock-infected pollen grains; sRNA density in HSVd-infected pollen grains. (B) The differential accumulation and distribution of the total reads of endogenous cucumber sRNAs ranging between 21 and 25 nt recovered from the mock- and HSVd-infected samples. (C) Relative increase of ribosomal-derived sRNAs (rb-sRNAs) and TE-derived sRNAs (te-sRNAs) in infected pollen: the ratio of reads obtained in infected pollen compared to the control library is shown. sRNAs derived from coding transcripts (cRNA) and centromeric regions (CRR) exhibit no significant alterations in the samples analysed. (D) Relative increase of rb-sRNAs (left) and te-sRNAs (right) in infected pollen compared to non-infected pollen based on sRNA-length. (This figure is available in colour at *JXB* online.)

In vegetative cells of cucumber HSVd infection induces a drastic change in the accumulation profile of rRNA-derived sRNAs that is associated with changes in the epigenetic regulation of those repeats (Martinez *et al.*, 2014; Castellano *et al.*, 2015, 2016). In order to evaluate whether HSVd infection induced global alterations in the epigenetic regulation of pollen, we analysed endogenous sRNAs from pollen of infected and non-infected plants (Fig. 3A). Viroid-derived sRNAs recovered from infected pollen were filtered out from this study. Approximately 85% of the 18–36 nt sRNAs reads (4 219 966 for HSVd-infected and 3 871 276 for mock samples) mapped with the cucumber genome. These reads were considered as endogenous pollen sRNAs and used in subsequent analysis. In HSVd-infected pollen there was a considerable increase of 21, 23, 24, and 25 nt sRNAs (Fig. 3B). In contrast, 22 nt sRNAs were present at similar frequencies. These results indicate that HSVd accumulation in pollen grains causes changes in endogenous sRNAs, resembling – at least in part – those observed in HSVd-infected cucumber vegetative tissues.

When endogenous sRNAs recovered from infected and healthy pollen grains were analysed by pairwise alignment against different *Cucumis sativus* transcript categories (Li *et al.*, 2011), we observed that ribosomal and TE-derived sRNAs were significantly over-accumulated in HSVd-infected pollen grains (Fig. 3C, Supplementary Figs S3 and S4). This observation was validated by analysing the accumulation of representative sRNAs derived from rRNA and TE transcripts by stem-loop qRT-PCR (see Supplementary Fig. S5). In contrast, no significant differences were obtained when sRNAs derived from the coding and centromeric regions of the cucumber genome were analysed (Fig. 3C). A more detailed analysis of up-regulated sRNA classes showed that their over-accumulation in infected samples was detectable in all size classes (21 to 25 nts) (Fig. 3D). Together, these results indicate that HSVd accumulation in pollen grains is associated with alterations in endogenous sRNA levels, mainly affecting the accumulation of sRNAs derived from repetitive regions of the genome.

### Viroid infection modifies cytosine methylation of repetitive regions in pollen

To investigate if the increase in sRNAs derived from rDNA and TEs observed in infected pollen could be linked to alterations in the host epigenetic landscape, we analysed the methylation pattern of representative regions of the repetitive pollen DNAs exhibiting sRNA-derived over-accumulation. Specifically, we analysed the rRNA promoter region previously described by Martinez *et al.* (2014) and a TE region that had the highest increase in sRNA accumulation. The genomic DNA extracted from pollen grains of HSVd-infected and mock-inoculated cucumber plants was bisulfite-converted and subjected to PCR to amplify specific regions of the rDNAs and TE DNA.

**Fig. 4.**
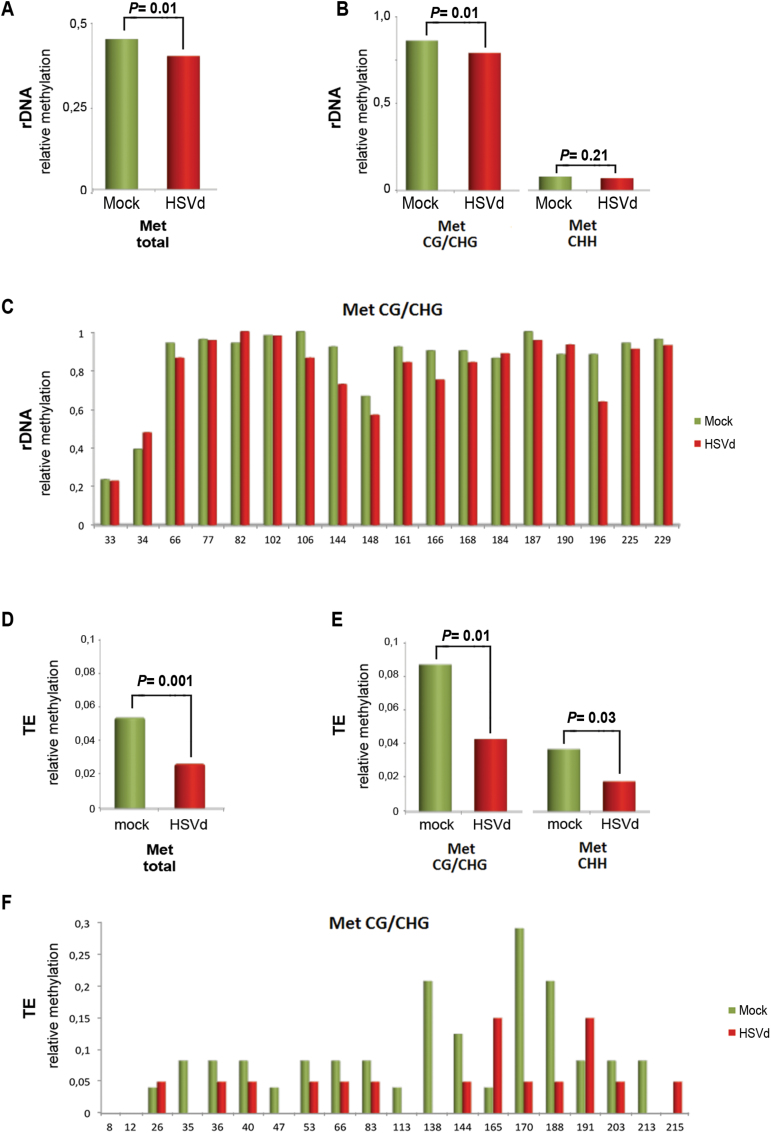
HSVd infection affects the methylation patterns of rRNA genes and TE in pollen grains. (A) The relative (HSVd/Mock) total rDNA methylation levels. Total methylation means are 0.40 (mock) and 0.37 (HSVd). Statistical differences were determined using a paired *t*-test. (B) Analysis of symmetric and asymmetric cytosine methylation levels in analysed samples of rDNA. Symmetric methylation means are 0.89 (mock) and 0.83 (HSVd). Asymmetric methylation means are 0.06 (mock) and 0.05 (HSVd). Statistical differences were determined using a paired *t*-test. (C) Position-specific relative methylation levels in CG and CHG contexts in the analysed samples of rDNA. (D) Relative (HSVd/Mock) total TE methylation. Total methylation means are 0.055 (mock) and 0.022 (HSVd). Statistical differences were determined using a paired *t*-test. (E) Analysis of symmetric and asymmetric cytosine methylation in the analysed samples of TEs. Symmetric methylation means are 0.084 (mock) and 0.041 (HSVd). Asymmetric methylation means are 0.037 (mock) and 0.018 (HSVd). Statistical differences were determined using a paired *t*-test. (F) Position-specific relative methylation levels in CG and CHG contexts in the analysed TEs. (This figure is available in colour at *JXB* online.)

We analysed a specific promoter sequence of 201 nt located between positions –80 and +121 of the 45S-rDNA containing 16 symmetric (12 CG, 4 CHG) and 23 asymmetric (CHH) potential methylation sites (see Supplementary Fig. S6A, B). PCR products were cloned, and the sequences of 51 and 44 clones were compiled for control and infected samples, respectively. Methylation analysis revealed that HSVd infection resulted in a significant decrease in the relative number of total methylated cytosine residues when compared to the control (Fig. 4A). Hypomethylation was restricted to symmetric (CG/CHG) sequence contexts (Fig. 4B, C), and not detected at asymmetric (CHH) positions (Fig. 4B). We further analysed a 236-bp region of a TE with homology to a Copia element (termed *cuc_reannotTE.Scaffold000159.7* in the reannotation of TEs from Li *et al.*, 2011) containing 20 symmetric (9 CG, 11 CHG) and 41 asymmetric (CHH) potential methylation sites (see Supplementary Fig. S7A). Similar to the 45S-rDNA locus, HSVd infection resulted in a significant reduction in the relative number of methylated cytosine residues of this transposable DNA compared to the mock-inoculated controls (Fig. 4D). This drastic hypomethylation affected both symmetric and asymmetric sequence contexts (Fig. 4E, F) Hypomethylation of the Copia element in the symmetric sequence context was also observed in vegetative tissues of HSVd-infected cucumber plants (see Supplementary Fig. S6B, C), revealing that this effect was not restricted to pollen. Taken together, these results indicate that in pollen grains, similar to vegetative tissues, rDNA and TEs lose DNA methylation in response to viroid infection, reinforcing the close interplay suggested to exist between HSVd-pathogenesis and host-epigenetic alterations in these classes of repetitive DNAs.

### HSVd infection promotes transcriptional alterations in the pollen grain

Silencing of repetitive DNA is a self-reinforcing transcriptional regulatory phenomenon mediated by siRNA-directed cytosine methylation and heterochromatin formation (Slotkin and Martienssen, 2007; Law and Jacobsen, 2010) that is dynamically regulated during plant development and stress (Martinez and Slotkin, 2012). To investigate whether the observed increase of sRNAs derived from rRNA and TEs and DNA hypomethylation could be associated with alterations in the host transcriptional activity, we analysed the accumulation of transcripts derived from these regions.

**Fig. 5.**
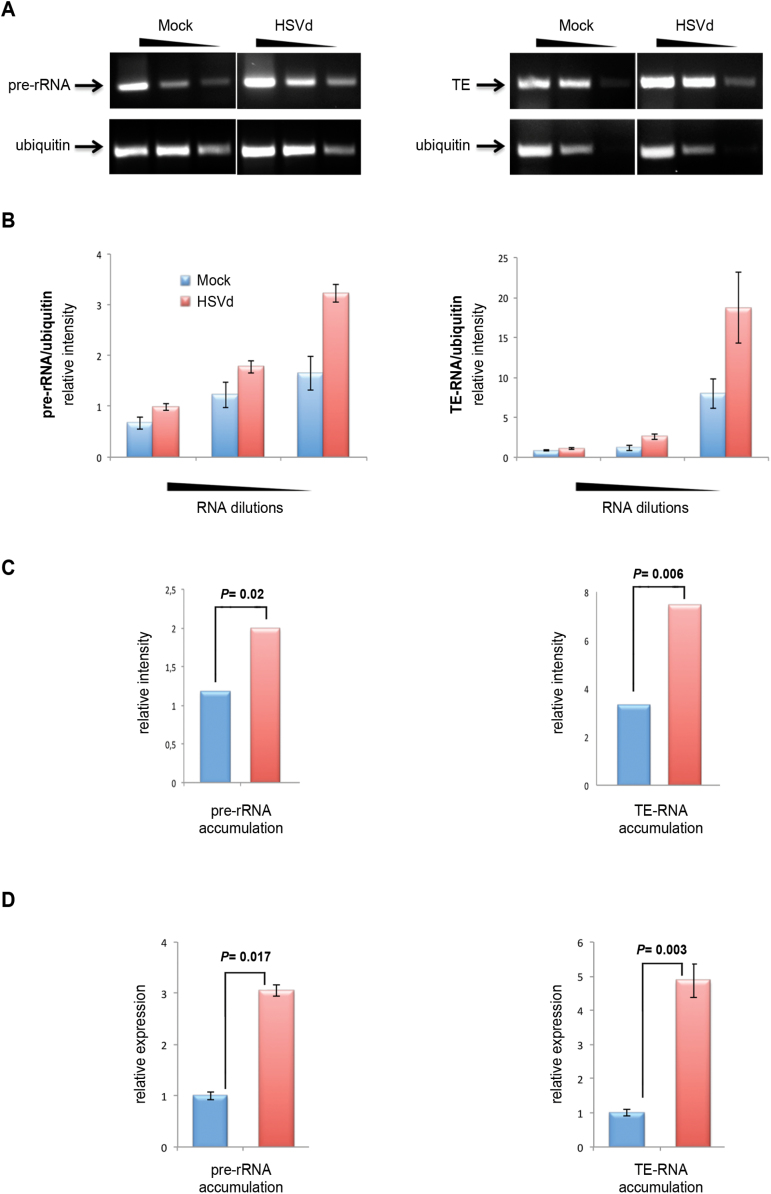
Differential accumulation of the precursor for rRNAs (pre-rRNAs) and TE-derived transcripts in infected pollen. (A) Representative RT-PCR analysis of the pre-rRNA (left) and TE (right) expression in serial dilutions (500, 100, and 20ng) of HSVd-infected and control total RNAs. RT-PCR amplification of ubiquitin mRNA served as a normalization control. (B) The relative accumulation (in relation to ubiquitin expression) of pre-rRNA (left) and TE-derived transcripts (right) in the serial dilutions shown in (A), determined by measurement of the band intensity, which was measured using the Image-J application (https://imagej.nih.gov/ij/). Error bars represent the standard error. (C) Comparison of relative pre-RNA and TE transcript accumulation (estimated from the sum of the intensity of the RT-PCR products) in control and infected pollen grains. The data are mean values obtained for pre-rRNA and TE amplification relative to the ubiquitin normalization control. Statistical significance was tested using a paired *t*-test. (D) Comparison of relative pre-RNA and TE transcript accumulation in control (and HSVd-infected pollen grains estimated by RT-qPCR analysis relative to the ubiquitin normalization control. The results shown are the means of three replicates. Error bars represent the standard error. Statistical significance was tested using a paired *t*-test. (This figure is available in colour at *JXB* online.)

To do this, a primer complementary to the 3′ end of the internal transcribed spacer 2 (ITS2-A) of the 45S rRNA transcription unit (see Supplementary Fig. S6C) was employed to generate the cDNA template. The pair 5.8s-Fw/5.8s-Rv was used to differentially amplify by PCR the unprocessed rRNA. We observed significantly increased levels of pre-rRNA and TE-derived transcripts in pollen RNA from viroid-infected plants compared to the mock-inoculated controls (Fig. 5A–C). Similar results were obtained when the accumulation of both pre-rRNA and TE-derived transcripts in control and HSVd-infected pollen grains was analysed by real-time quantitative PCR (Fig. 5D). We thus conclude that HSVd-infection promotes increased transcriptional activity of the repetitive DNAs in pollen that were analysed, revealing that the viroid-induced hypometylation causes changes in the transcriptional status in cucumber reproductive tissues.

## Discussion

Drastic alterations of the epigenome in response to pathogen attack have been described for both plants (Agorio and Vera, 2007; Boyko *et al.*, 2007, 2010; Lopez *et al.*, 2011; Dowen *et al.*, 2012; Luna and Ton, 2012; Yu *et al.*, 2013) and animals (Tang *et al.*, 2011). These observations support the notion that epigenetic reprogramming of transcriptional activity is a general mechanism controlling the host response to pathogen infection (Zhu *et al.*, 2016). Consistent with this idea, HSVd accumulation in vegetative tissues causes hypomethylation of the promoter region of rRNA genes, leading to significant alterations in rRNA transcription (Martinez *et al.*, 2014; Castellano *et al.*, 2015). In this study we show that both viroid mature forms and vd-sRNAs accumulate in pollen grains of HSVd-infected plants, indicating that during the pathogenesis process the viroid is able to invade this reproductive cell. We furthermore demonstrate that general sRNA profiles are altered in this reproductive tissue; in particular, we observed increased sRNAs derived from rRNA and TE transcripts. Increased levels of ribosomal RNA-derived sRNAs (rb-sRNAs) were previously reported to occur in leaves of cucumber and *N. benthamiana* plants infected by HSVd, reinforcing the close interplay between viroid-induced pathogenesis and host rRNA metabolism. Importantly, the observation that sRNAs derived from TEs (te-sRNAs) are also over-accumulated in infected tissue suggests that the viroid-induced transcriptional alteration is a more general phenomenon that is not restricted only to rRNA repeats. In line with this possibility, bisulfite sequencing clearly correlated HSVd infection with changes in DNA methylation – in a symmetric sequence context – in both rDNA and TE.

The hypomethylated status of the rDNA regions analysed is consistent with the significant size increase of the nucleolus in the generative nucleus of HSVd-infected pollen. The nucleolus is mainly composed of active and inactive rDNA (Lam and Trinkle-Mulcahy, 2015), and consequently it is reasonable to suppose that changes in nucleolar morphology (Fig 1D) may be associated with alterations in the rRNA transcriptional activity observed in pollen grains during viroid infection (Fig. 5). This resembles, at least in part, the observation that in soybean plants grown at low temperature different condensation states of nucleololar chromatin (which occurred in response to the stress) correlated with changes in DNA methylation levels and transcriptional activity (Stepiński, 2013). Moreover, and considering that the heterochromatin of the nucleus is mostly occupied by TEs and other repetitive DNAs (Lippman *et al.*, 2004), it seems likely that HSVd infection causes a general reduction of DNA methylation at repeat regions. Interestingly, decondensation of centromeric regions and rDNA loci also occurs in response to heat stress (Pecinka *et al.*, 2010), suggesting a general stress response in heterochromatin. Whether this is connected to increased metabolic activities that require increased ribosome production remains to be investigated. Interestingly, we observed that HSVd-infected pollen had an increased germination rate compared to pollen from mock-infected plants, which may be a consequence of increased ribosomal activity.

Together, our data support the idea that HSVd accumulation in pollen grains promotes host epigenetic alterations, as evidenced by drastic changes in rDNA and TE expression. Although the molecular basis of this phenomenon remains to be fully elucidated, it was recently shown that in infected leaves HSVd RNA is able to bind and functionally subvert host HDA6, promoting rDNA hypomethylation (Castellano *et al.*, 2016). HDA6 acts as an epigenetic regulator required to confer memory of the silent state and to maintain DNA methylation (Aufsatz *et al.*, 2002; Probst *et al.*, 2004; Earley *et al.*, 2010; Liu *et al.*, 2012; Blevins *et al.*, 2014; Hristova *et al.*, 2015). As we observed similar changes in DNA methylation in pollen and vegetative tissues, this suggests that HSVd also impairs the function of HDA6 in pollen. However, in contrast to HSVd-infected cucumber leaves where 21-nt and 24-nt rb-sRNAs were respectively up- and down-regulated, the methylation loss in pollen grains was accompanied by a general increase of sRNAs in all size classes. An increase of 21-nt TE or rRNA-derived sRNAs is a known characteristic of Pol II transcription of repetitive regions upon down-regulation of epigenetic factors such as DDM1 or HDA6 (Earley *et al.*, 2010; McCue *et al.*, 2012). Consequently, our results suggest that although the altered epigenetic scenario is similar in both vegetative and reproductive tissues, specific aspects of the HDA6-dependent regulatory mechanisms underlying this phenomenon differ. In this regard, recent data have demonstrated that DNA methylation can also be modulated by DCL-independent siRNAs (Yang *et al.*, 2016; Dalakouras *et al.*, 2016; Ye *et al.*, 2016). These siRNAs (ranging from 20 to 60 nt in length) are mainly derived from repetitive sequences and loaded in AGO4 to direct DNA methylation (Ye *et al.*, 2016). Further studies are needed in order to determine if this non-canonical regulatory pathway could be also affected in HSVd-infected pollen grains.

Furthermore, it will be important to test whether epigenome changes in response to HSVd infection have functional consequences in the next generation of plants. Thus far, transgenerational effects of stress are rather questionable (Pecinka and Scheid, 2012); however, our data show a stress response affecting the chromatin status of the generative nucleus, suggesting that these changes could potentially be inherited to the next generation.

In summary, we have shown that HSVd infection induces hypomethylation of rRNA genes and TEs in pollen grains, providing the first example of epigenome changes in reproductive cells upon pathogen infection.

## Supplementary data

Supplementary data are available at *JXB* online.

Figure S1. Validation of HSVd-infected cucumber plants.

Figure S2. PCR of total RNA extracts in order to discard DNA contamination.

Figure S3. Analysis of differentially expressed ribosomal-derived sRNAs in infected pollen.

Figure S4. Analysis of TE-derived sRNAs differentially expressed in infected cucumber pollen.

Figure S5. Validation by stem-loop qRT-PCR of representative sRNAs highly accumulated in HSVd-infected pollen grains.

Figure S6. Diagram of the rDNA intergenic region analysed by bisulfite sequencing.

Figure S7. HSVd infection affects the methylation patterns of TE DNA in cucumber leaves.

Table S1. Description of primers used in the qRT-PCR assays of rRNA and TE transcripts.

Table S2. Description of primers used in stem-loop qRT-PCR assays.

Supplementary Data
