# Supplementary material for: Changes in the DNA methylation pattern of the host male gametophyte of viroid-infected cucumber plants
Source: J Exp Bot. 2016 Oct 3;67(19):5857–68. doi: 10.1093/jxb/erw353 (PMC5066502; doi:10.1093/jxb/erw353)
Supplement: Supplementary Data [file supp_67_19_5857__index.html]

Changes in the DNA methylation pattern of the host male gametophyte of viroid-infected cucumber plants — Changes in the DNA methylation pattern of the host male gametophyte of viroid-infected cucumber plants — Supplementary Data 

# Changes in the DNA methylation pattern of the host male gametophyte of viroid-infected cucumber plants

## Supplementary Data

Data files

- supplementary\_figures\_S1\_S7\_tables\_S1\_S2.pdf - Supplementary Data
