# Supplementary material for: Changes in the DNA methylation pattern of the host male gametophyte of viroid-infected cucumber plants
Source: J Exp Bot. 2016 Oct 3;67(19):5857–68. doi: 10.1093/jxb/erw353 (PMC5066502; doi:10.1093/jxb/erw353)
Supplement: Supplementary Data [file supp_erw353_supplementary_figures_S1_S7_tables_S1_S2.pdf]

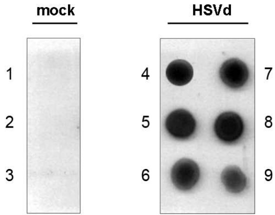

Supplementary Figure S1: Identification of HSVd-infected cucumber plants: Detection of HSVd-RNA by dot-blot hybridization in apical leaves of cucumber plants used to pollen extraction. Total RNAs extracted from three (1 to 3) mock-inoculated and six (4 to 9) HSVd-inoculated cucumber plants were dotted onto positively charged Nylon membranes. Hybridization assays was performed, using as probe, a Digoxigenin-labelled HSVd negative-strand specific RNA followed of chemiluminescent detection, as previously described (Gomez and Pallas, 2006).

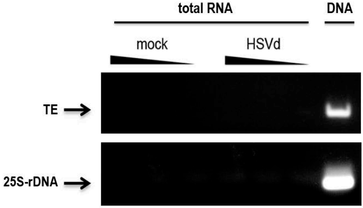

Figure Supplementary 2: Representative PCR analysis in serial dilutions of HSVd-infected and control total RNA extracts to corroborate the absence of DNA in RNA samples used to analysis of pre-rRNA and TE-derived transcripts expression. Extracts of total DNAs were used as positive amplification controls.

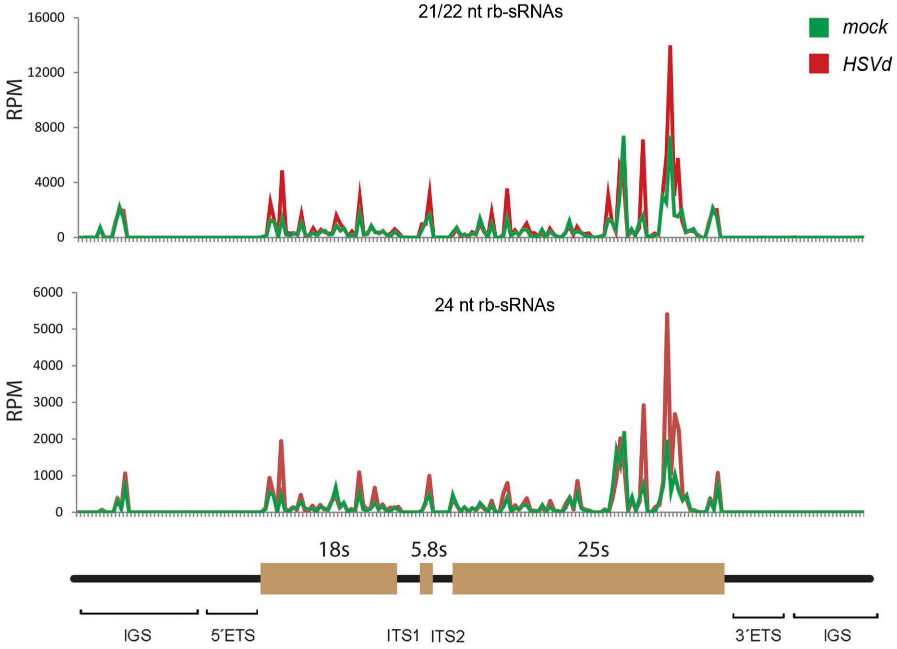

Figure Supplementary 3: Analysis of differentially expressed ribosomal-derived sRNAs in infected pollen grains. The rb-sRNAs (21/22 nt lengh upper, and 24 nt lower panel) recovered from the infected (red lines) or the mock-inoculated plants (green lines) were plotted according to the position of their 5´-end onto the cucumber rRNA sequence. The values on the Y-axis represent the number of normalized reads in each library. Diagram (no scale) of the rRNA unit. IGS (Non transcribed intergenic spacer). The external (ETS) and internal (ITS) transcribed spacers are removed during rRNA processing.

A

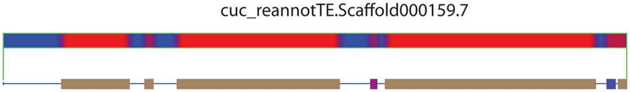

Name	From	To	Name	From	To	Class	Dir	Sim	Pos/Mm:Ts	Score
31229.tmp/data.ori	472	1024	Copia-74_GM-I	171	723	LTR/Copia	d	0.6435	1.9767	650
31229.tmp/data.ori	1152	1218	Copia-56_VV-I	861	926	LTR/Copia	d	0.7612	2.5000	257
31229.tmp/data.ori	1406	2722	Copia-34_NN-I	1157	2473	LTR/Copia	d	0.6927	1.9205	2620
31229.tmp/data.ori	2975	3023	DNA-8-1_DR	394	453	DNA	d	0.8462	1.6667	219
31229.tmp/data.ori	3094	4788	Copia-34_NN-I	2824	4524	LTR/Copia	d	0.7264	2.0145	5510
31229.tmp/data.ori	4876	4946	X1_LINE	234	310	NonLTR/CR1	c	0.7333	1.4545	229
31229.tmp/data.ori	4975	5039	Copia-15_GR-LTR	263	323	LTR/Copia	d	0.7969	1.6000	237

B

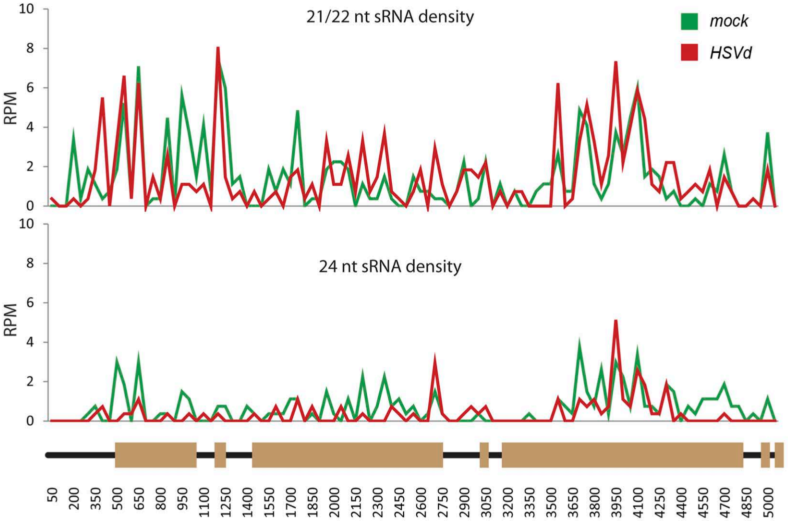

Figure Supplementary 4: Analysis of TE-derived sRNAs differentially expressed in infected cucumber pollen grains. (A) Screenshot of the CENSOR (http://www.girinst.org/censor/index.php) sequence homology analysis of the TE cuc_reannotTE.Scaffold000159.7. The analysis reveals homology of the element with different members of the Copia family. (B) sRNA accumulation profile over the TE cuc_reannotTE.Scaffold000159.7 in mock-inoculated (green line) and HSVd-infected (red line) pollen grains. Densities of 21/22 (upper panel) and 24 (lower panel) nt sRNAs are shown. Reads were normalized to reads per million (RPM).

A

	Analyzed sRNAs				
			total reads		
	Sequence	lenght	HSVd(+)	Mock	*LogFC*
**rRNA-derived**					
rb-sRNA	ATTTCACAAAGTCGGACTAGA	21	1938	182	3,41256
**TEs-derived**					
TE-sRNA	AGCCCGATCGTCCCTAGAAGAAGT	24	155	3	5,69116
**control**					
miRNA159-a	TTTGGATTGAAGGGAGCTCTA	21	604	575	0,07099

B

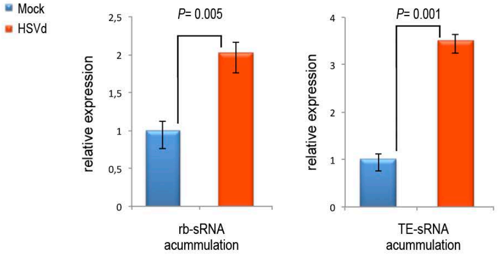

Supplementary Figure S5: Validation by stem-loop qRT-PCR of representative sRNAs highly accumulated in HSVd-infected pollen grains. (A) Description of the representative differentially expressed sRNAs derived from rRNA and TEs-transcripts, selected for qRT-PCR quantification. Information about miR159 control is also included. (B) Comparison of relative accumulation of sRNAs described in (A) in mock-control (blue bars) and HSVd-infected (red bars) pollen grains estimated by RT-qPCR analysis. The values are relative to the normalization control miR-159a. The results shown are the means of three replicates. Error bars represent standard error. Statistical significance was tested using a paired t-test. P values are shown in the figure. The primers used are detailed in the Table S1.

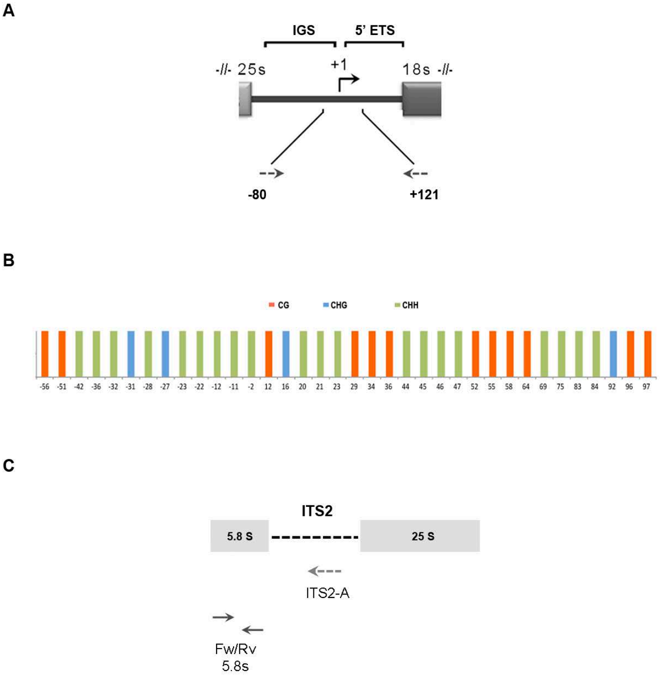

Figure Supplementary 6: (A) Diagram (no scale) of the rDNA intergenic region highlighting the promoter zone analyzed by bisulfite sequencing. The arrows represent the oligos used in the PCR assay and their relative position in the rDNA. IGS: Intergenic spacer, ETS: external transcribed spacer. +1 represent the transcription initiation site. (B) Graphic representation of the potential symmetric (CG – red bars - and CHG – blue bars) and asymmetric (CHH – green bars) positions predicted to exist within the analyzed rDNA region. (C) Diagram (no scale) of the partial pre-27S rRNA (ITS2 refer to unprocessed internal transcribed spacers). The dotted arrow below depicts the oligo used for the RT reaction starting from the ITS2 region of the pre-rRNA. Solid arrows indicate the oligos used in the PCR amplification.

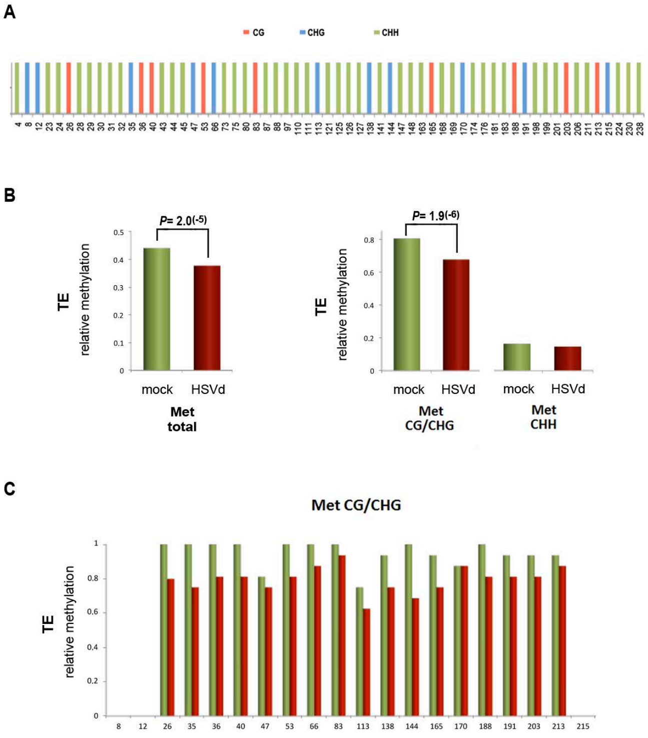

Figure Supplementary 7: HSVd infection affects the methylation patterns of TE DNA in cucumber leaves. (A) Graphic representation of the potential symmetric (CG – red bars - and CHG – blue bars) and asymmetric (CHH – green bars) positions predicted to exist within the analyzed TE DNA region. (B) left panel: Histogram documenting the total methylation levels in TE DNA obtained from mock and HSVd-infected cucumber leaves. Right panel: Schematic representation of both symmetric and asymmetric cytosine methylation levels in analysed TE DNA. Statistical significance was tested using a paired t-test. Only significant P values are shown in the figure. (C) Positions of methylcytosines in the analysed TE DNA, displayed in symmetric (CG and CHG) context. The height of the bar represents the frequency at which a cytosine was methylated.

Table S1: Description of primers used in qRT-PCR assays of rRNA and TE transcripts..

Primer name	Sequence	Length	GC%	Melting Temp.
qPCR-TE-Rv	AGACGAGAGGCATTCTGACG	20	55	56ºC
qPCR-TE-Dir	GCTTTCGGGAAATACCTCGC	20	55	57ºC
qPCR-UB-DIR	GATTTTCGCCGGAAAGCAGC	20	55	57ºC
qPCR-UB-REV	TGTAGAACTGAAGGACGGCG	20	55	56ºC
qPCR-25S-DIR	TGGATGTCCCCAACACAATCC	21	52	57ºC
qPCR-25S-REV	CCCATGTGGACTGATCGCC	19	63	58ºC

Table S2: Description of primers used in stem-loop qRT-PCR assays. sRNA specific sequence in marked in bold in RT-Primer sequences.

**siR-rRNA-1**	SEQUENCE	ATTTCACAAAGTCGGACTAGA
	RT-PRIMER	GTTGGCTCTGGTGCAGGGTCCGAGGTATTCGCACCAGAGCCAAC**TCTAGTC**
	FW PRIMER	CGCCGC**ATTTCACAAAGTCG**
**siR-TE-1**	SEQUENCE	AGCCCGATCGTCCCTAGAAGAAGT
	RT-PRIMER	GTTGGCTCTGGTGCAGGGTCCGAGGTATTCGCACCAGAGCCAAC**ACTTCTTC**
	FW PRIMER	TAGCAGC**AGCCCGATCGTCCC**
**miR-159a**	SEQUENCE	TTTGGATTGAAGGGAGCTCTA
	RT-PRIMER	GTTGGCTCTGGTGCAGGGTCCGAGGTATTCGCACCAGAGCCAAC**TAGAGC**
	FW PRIMER	GCGCC**TTTGGATTGAAGGGAG**
**Rv universal primer**	GTGCAGGGTCCGAGGTATTC	
